# Frequency and phenotype consequence of *APOC3* rare variants in patients with very low triglyceride levels

**DOI:** 10.1186/s12920-018-0387-1

**Published:** 2018-09-14

**Authors:** Dana C. Crawford, Nicole A. Restrepo, Kirsten E. Diggins, Eric Farber-Eger, Quinn S. Wells

**Affiliations:** 1 Department of Population and Quantitative Health Sciences, Institute for Computational Biology, Case Western Reserve University, 2103 Cornell Road, Wolstein Research Building, Suite 2-527, Cleveland, OH 44106 USA; 2 Cancer Biology, Vanderbilt University School of Medicine, Nashville, TN USA; 3 Vanderbilt Institute for Clinical and Translational Research, Vanderbilt University Medical Center, Nashville, TN USA; 4 Departments of Medicine and Pharmacology, Vanderbilt University Medical Center, Nashville, TN USA

**Keywords:** Precision medicine, Triglycerides, Biobank, Electronic health records, *APOC3*

## Abstract

**Background:**

High levels of triglycerides (TG ≥200 mg/dL) are an emerging risk factor for cardiovascular disease. Conversely, very low levels of TG are associated with decreased risk for cardiovascular disease. Precision medicine aims to capitalize on recent findings that rare variants such as *APOC3* R19X (rs76353203) are associated with risk of disease, but it is unclear how population-based associations can be best translated in clinical settings at the individual-patient level.

**Methods:**

To explore the potential usefulness of screening for genetic predictors of cardiovascular disease, we surveyed BioVU, the Vanderbilt University Medical Center’s biorepository linked to de-identified electronic health records (EHRs), for *APOC3* 19X mutations among adult European American patients (> 45 and > 55 years of age for men and women, respectively) with the lowest percentile of TG levels. The initial search identified 262 patients with the lowest TG levels in the biorepository; among these, 184 patients with sufficient DNA and the lowest TG levels were chosen for Illumina ExomeChip genotyping.

**Results:**

A total of two patients were identified as heterozygotes of *APOC3* R19X for a minor allele frequency (MAF) of 0.55% in this patient population. Both heterozygous patients had only a single mention of TG in the EHR (31 and 35 mg/dL, respectively), and one patient had evidence of previous cardiovascular disease.

**Conclusions:**

In this patient population, we identified two patients who were carriers of the *APOC3* 19X null variant, but only one lacked evidence of disease in the EHR highlighting the challenges of inclusion of functional or previously associated genetic variation in clinical risk assessment.

**Electronic supplementary material:**

The online version of this article (10.1186/s12920-018-0387-1) contains supplementary material, which is available to authorized users.

## Background

Personalized or precision medicine is meant to distinguish tailored treatment from trial and error. The concept of precision medicine is not new and has been in practice arguably since the dawn of modern medicine [1]. Health care providers have long collected detailed data on patients, ranging from basic personal histories to technical laboratory assays and diagnostic procedures, to provide specific diagnoses and treatments. These tools ordered in the precision medicine setting are constantly evolving, for example, the evolution of myocardial infarction diagnosis [2], resulting in high resolution and, in some cases, highly predictive individualized data.

Today’s concept of precision medicine has evolved to specifically include the genetic profile of a patient in the prevention, diagnosis, and treatment of disease [3, 4]. Previous proxies for genetic profiles such as sex, race/ethnicity, family history, and response to therapy are now being augmented by Clinical Laboratory Improvement Amendments-certified genotyping and sequencing at both the targeted and whole genome level. Indeed, technological advances in high-throughput genomics coupled with their rapid decreases in costs have made generating the data almost trivial, and the emergence of electronic health records (EHRs) in part through the HITECH Act [5, 6] make it possible to effectively deliver personalized medicine to the patient.

A major challenge in the delivery of personalized medicine is not the collection of data, but the interpretation of the data. Large-scale population sequencing studies have demonstrated that potentially functional variants exist in all DNA samples sequenced, but the biological and statistical data lag in filtering the potential from truly functional, and even less data are available to direct the course of clinical action based on these genotypes. As such, major areas of active research focus on methods to translate genomic discoveries into clinical applications [7]. This broad area of investigation includes research on who to test, what to test, how to test, what to report, how to report, and how to measure its effectiveness, to name a few.

To begin to address some of these gaps in research, we have undertaken a pilot study of genotyping for a loss of function variant, *APOC3* R19X (rs76353203), in a targeted population of 184 European American adults (men > 45 and women > 55 year of age) with very low triglyceride levels in BioVU, the Vanderbilt University Medical Center (VUMC)‘s biorepository linked to de-identified EHRs. Triglyceride levels (TG) are a common biomarker measured in the clinic, and patients with extreme triglyceride (TG) levels may be flagged for further evaluation for cardiovascular disease risk assessment (TG ≥200 mg/dL). Recent studies have identified a loss-of-function variant in *APOC3* (R19X or rs76353203) associated with low triglyceride levels and diminished post-prandial lipemia in heterozygous carriers [8] and improved clearance of plasma TGs after a fatty meal in homozygous carriers [9]. Although rare in the general population [10], we hypothesized that the loss-of-function allele would be at an increased frequency in this extreme population. Based on previous reports, we also hypothesized that evidence of cardiovascular disease would be absent in EHRs of these *APOC3* 19X carriers with very low TG levels.

## Methods

### Study population

The study population presented here is from BioVU, the VUMC’s biorepository linked to de-identified EHRs. BioVU operations [11] and ethical oversight [12] have been previously described. Briefly, DNA is extracted from discarded blood drawn for routine clinical care at Vanderbilt outpatient clinics in Nashville, Tennessee and surrounding areas. The DNA samples are linked to a de-identified version of the patient’s EHR. The data in this study were de-identified in accordance with provisions of Title 45, Code of Federal Regulations, part 46 (45 CFR 46); therefore, this study was considered non-human subjects research by the Vanderbilt University Internal Review Board.

### Phenotyping

The de-identified EHR contains both structured (International Classification of Diseases, Ninth Revision, Clinical Modification (ICD-9-CM) billing codes; current procedural terminology (CPT) codes; problems lists; labs) and unstructured (clinical free text) data accessible for electronic phenotyping. We extracted all available labs for triglyceride levels in September 2012 for European American adults (> 45 years of age of men and > 55 years of age for women) and examined individuals whose median TG levels constituted the lowest 1% of BioVU at the time of data extraction. A total of 262 individuals were identified. Manual review of the clinical text for 30 random patients with low TG levels prior to selection for genotyping failed to identify obvious documented diagnoses or notes that may have led to very low TG levels in these patients. From these 262 individuals, 184 were selected for Illumina HumanExome BeadChip genotyping based on DNA quality and quantity, and preference was given to individuals with more than one triglyceride level reflecting consistently low TG levels.

The de-identified EHRs for the 184 patients genotyped on the Illumina HumanExome BeadChip were re-examined in July 2015 for evidence of myocardial infarction, revascularization, and other heart disease. Myocardial infarction was defined by mention of ICD-9-CM codes 410 or 410.* or a problem list mention of “MI” or “myocardial infarction.” Revascularization was defined by CPT codes (Table 1). Other heart disease was defined as a problem list mention of “coronary artery disease,” “CAD,” “coronary heart disease,” or “CHD.”

**Table 1:** Current Procedural Terminology codes used to define revascularization among European American patients in BioVU with very low triglyceride levels

CPT code	Description
33510–33514; 33516	Coronary artery bypass grafting with venous grafting only (1–6 or more grafts)
33515	Coronary artery bypass (old code)
33517–33519; 33521–33523	Coronary artery bypass grafting with venous and arterial grafting (1–6 or more vein grafts); billed in conjunction with 33533–33536 (33517–33523 cannot be billed alone).
33520	Coronary artery bypass (old code)
33534–33536	Coronary artery bypass grafting with arterial grafting only (2–4 or more grafts)
92980–92981	Transcatheter placement of an intracoronary stent, percutaneous, with or without other therapeutic intervention, any method (single vessel and each additional vessel)
92982; 92984	Percutaneous transluminal coronary balloon angioplasty (single vessel and each additional vessel)
92995; 92996	Percutaneous transluminal coronary atherectomy, by mechanical or other method, with or without balloon angioplasty (single vessel and each additional vessel)

### Genotyping and statistical methods

Vanderbilt Technologies for Advanced Genomics (VANTAGE) genotyped 184 BioVU samples and eight International HapMap reference samples using the Illumina HumanExome BeadChip (v1.0 for 48 samples and v1.2 for 144 samples). As recommended by other research groups with Exome BeadChip experience [13], genotypes for these 192 samples were called as part of larger, ongoing Exome BeadChip projects in BioVU genotyped by VANTAGE. *APOC3* R19X (rs76353203/exm957809) was directly assayed by the Exome BeadChip, and we extracted these called genotypes for further analysis.

We also extracted genotypes for SNPs previously associated with incident myocardial infarction in European Americans [14]. Of the 46 previously associated SNPs, 37 were directly assayed by the Exome BeadChip (Additional file 1: Table S1). We then calculated both unweighted and weighted genetic risk scores (GRS) based on the genotypes of these 37 SNPs. Unweighted GRS were calculated by counting the number of risk alleles per individual. Weighted GRS were calculated based on counts of risk alleles multiplied or weighted by odds ratios recently reported for European American cases of coronary artery disease in comparison with controls [15].

## Results

As detailed in the Methods section, 262 European American men > 45 and women > 55 years of age had the lowest TG levels in BioVU in 2012. Slightly less than half (44%) of the patient sample was female, and the average birth decade was the 1940s. Approximately half of the patients (53.1%) with the lowest TG levels had more than one TG available in the EHR. For those with more than one TG level, the median values were calculated. The overall median TG level in this lowest 1% was 36 mg/dL.

**Fig. 1:**
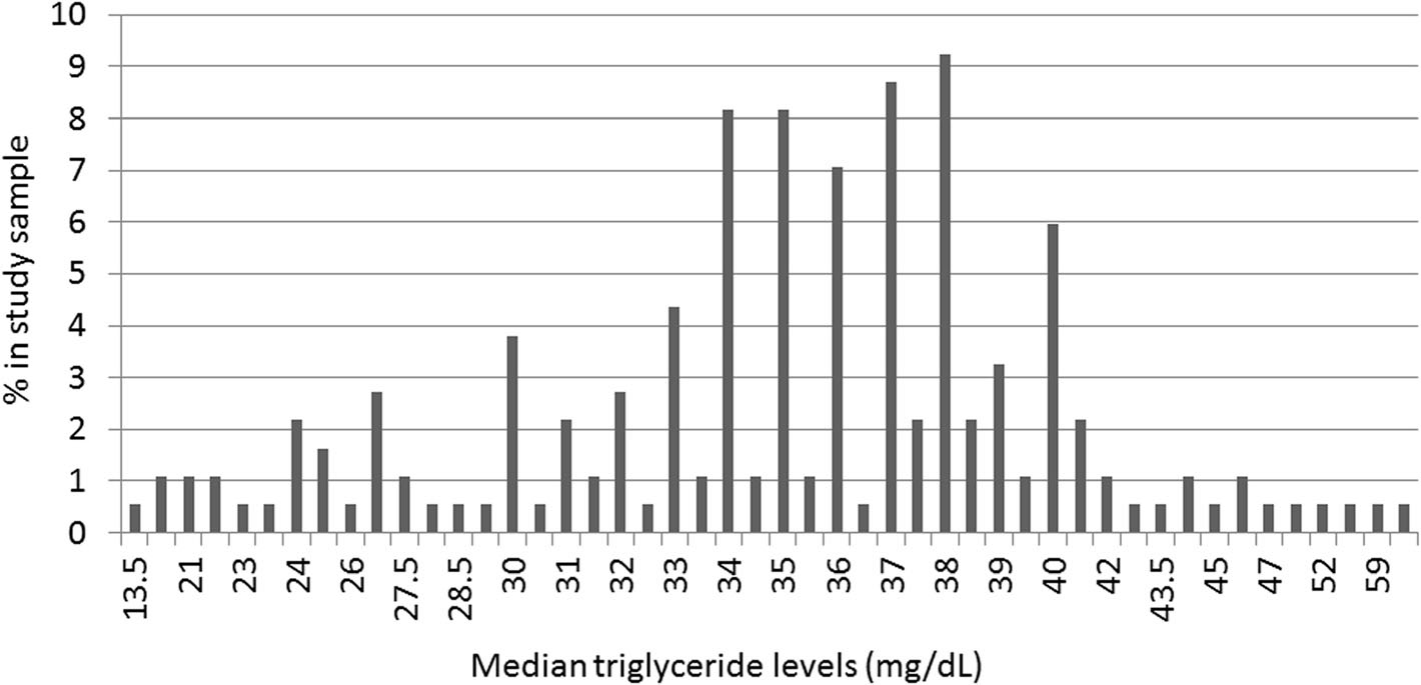
Distribution of low triglyceride levels among European American adults in BioVU. A total of 184 European American adults (men > 45 years and women > 55 years) with at least one triglyceride level in the lowest 1% of BioVU were genotyped on the Illumina HumanExome BeadChip for *APOC3* R19X. The frequency (expressed as percent in the study population) is given on the y-axis and the median triglyceride levels (in mg/dL) are given on the x-axis

The genotyped study population characteristics are given in Table 2. Like the 262 patients considered, the patients genotyped were majority male born in the 1940s and 1950s. The mean body mass index was 24.8 kg/m^2^, which is considered within the normal range. The first mention of TG in the clinical record was on average 39.3 mg/dL. The median of all TG levels available for this patient population was 36 mg/dL, ranging from 13.5 to 61 mg/dL (Fig. 1).

**Table 2:** Study population characteristics

Female, %	43.5
Decade of birth, %	
1910	0.5
1920	9.2
1930	14.7
1940	28.3
1950	37.5
1960	9.8
Mean (±SD) body mass index (kg/m^2^)	24.8 (±4.7)
Mean (±SD) first TG level (mg/dL)	39.3 (±18.4)

Study population characteristics (sex, decade of birth, average body mass index, and average first mention of triglyceride levels in patient’s electronic health record) are given for the *genotyped* 184 patients with the lowest 1% triglyceride levels in BioVU among European American men and women > 45 and > 55 years of age, respectively

*Abbreviations*: *SD* standard deviation, *TG* triglyceride

Among the 184 patients with low TG levels in BioVU genotyped here, 14.13%, 8.15%, and 12.5% had evidence in the EHR of a myocardial infarction, revascularization, and other heart disease, respectively. Only six out of 184 patients (3.26%) had evidence of all three. The unweighted and weighted GRS for patients with events (myocardial infarction or revascularization) or evidence for other heart disease were higher compared with patients without events or evidence for other heart disease, although these differences were not statistically significant (Table 3).

**Table 3:** Genetic risk scores, unweighted and weighted, by case status among European American patients with very low triglyceride levels

	All patients with very low TG levels (*n* = 184)	Patients with no evidence of MI, revascularization, or other heart disease (*n* = 144)	Patients with evidence of MI (*n* = 26)	Patients with evidence of revascularization (*n* = 15)	Patients with evidence of other heart disease (*n* = 23)	Patients with evidence of MI, revascularization, *and* other heart disease (*n* = 6)
Unweighted GRS	38.97 (3.36)	38.78 (3.36)	39.46 (3.47)	39.07 (3.95)	39.08 (3.57)	38.00 (4.38)
Weighted GRS	41.97 (3.63)	41.77 (3.63)	42.54 (3.72)	42.08 (4.32)	42.14 (3.82)	41.02 (4.84)

We calculated unweighted and weighted genetic risk scores (GRS) based on 37 SNPs and previous association estimates in European Americans with and without coronary artery disease (Additional file 1: Table S1). Unweighted GRS were calculated as counts of risk alleles per patient, and weighted GRS were calculated using the pooled odds ratios from CARDIoGRAM [15]. Shown are the means (standard deviations) for unweighted and weighted GRS for the total study population as well as by cases status. Although unweighted and weighted GRS were higher among all patient groups with events or other heart conditions compared with patients lacking evidence of events or other heart disease, these differences were not statistically significant (unpaired t-tests; *p* > 0.05)

We next examined the frequency of *APOC3* R19X (rs76353203) in this patient sample of extremely low TG levels. Among 182 patients successfully genotyped for this variant, two heterozygotes were identified for a sample allele frequency of 0.55%. The allele frequency for the loss-of-function allele (T) in this sample of very low TG levels is 6.9 to 27.5 fold higher compared with allele frequency estimates for American adults drawn from the general population [10, 16] and three to 10 fold lower in frequency compared with isolated populations [8, 17, 18] (Table 4).

**Table 4:** *APOC3* R19X frequency, by population

Population	Sample size	Carriers identified (Overall allele frequency)	Allele frequency in European-descent populations	PubMed ID or website
European American adults with very low triglyceride levels	184	2 (0.55%)	0.55%	- (present study)
Pennsylvania Amish	2503	140 (5.6%)	5.6%	19074352
Greek isolate	1219	48 (1.9%)	1.9%	24343240
Greek isolate	1087	34 (1.42%)	1.42%	27146844
Americans regardless of health status	19,613	31 (0.08%)	0.20%	25363704
Exome Aggregation Consortium (ExAC)	60,103	83 (0.07%)	0.046%	27535533 (http://exac.broadinstitute.org/ accessed May 2017)
National Heart, Lung, and Blood Institute (NHLBI) Grand Opportunity (GO) Exome Sequencing Project (ESP)	6495	3 (0.02%)	0.035%	24941081 (http://evs.gs.washington.edu/EVS/ accessed June 2015)
Ohio and Indiana Amish	1113	0 (−)	–	25363704
1000 Genomes Project	2500	0 (−)	–	(http://useast.ensembl.org/index.html accessed June 2015)
European Americans from Baltimore	214	0 (−)	–	19074352

Previous studies in outbred [10] and isolated [8, 17] populations suggest that the 19X mutation arose once on a single haplotype background. We therefore examined the diplotypes spanning *APOA1/C3/A4/A5* gene cluster assayed by the Illumina HumanExome Beachip for evidence of a single haplotype background containing the 19X mutation. Of the 78 SNPs assayed (spanning chr11: 116619073–116,707,837), seven SNPs overlapped with variants used to infer haplotypes in non-Hispanic whites of the Third National Health and Nutrition Examination Survey (NHANES III): rs28927680, rs964184, rs12286037, rs5110, rs675, rs5104, and rs76353203. We found that one 19X carrier was homozygous at all variants in this region that passed quality control except for 19X, and that this variant-containing haplotype background was similar to 19X backgrounds in NHANES III (G-C-C-C-A-T-T). In contrast, the second 19X carrier was heterozygous at eight sites including rs76343203. Interestingly, one of these eight heterozygous sites included rs138326449 (IVS2 + 1G > A), another *APOC3* rare variant associated with decreased levels of TG [16, 19, 20]. A query for rs138326449 in the 184 patients with very low triglycerides revealed an additional carrier for this variant. Both carriers of rs138326449 are, with the inclusion of this rare variant, heterozygous at more than one site, and one carrier has missing data (26/78 sites) at this gene cluster; therefore, his or her haplotypes could not be unambiguously inferred with confidence. Much like R19X, the frequency of rs138326449 (0.57%) is higher in this patient population of low TG levels compared with outbred population estimates [16, 19]. A third rare *APOC3* variant (rs147210663) associated with low TG levels [16] failed genotyping in this patient population.

**Fig. 2:**
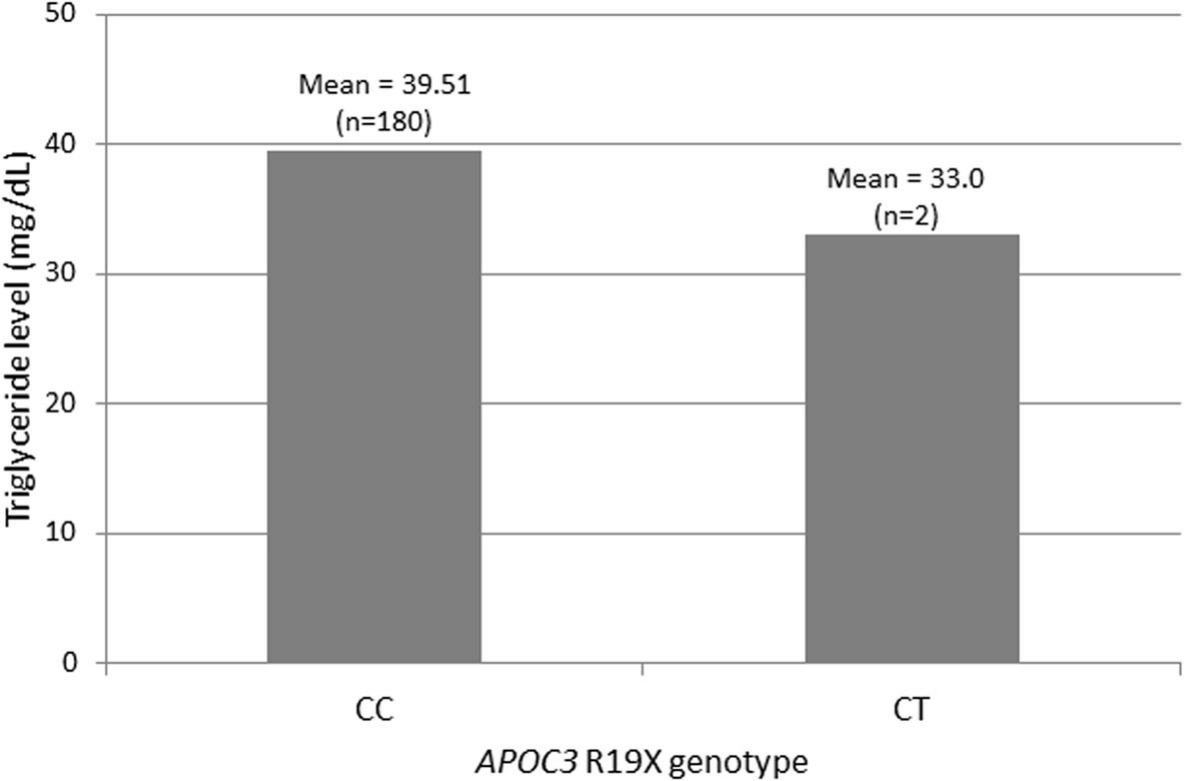
Mean triglyceride levels by *APOC3* R19X genotype. A total of 184 European American adults (men > 45 years and women > 55 years) with at least one triglyceride level in the lowest 1% of BioVU were genotyped on the Illumina HumanExome BeadChip for *APOC3* R19X. Two samples failed genotyping. The means for the first mentioned triglyceride level (y-axis) were calculated for the non-carriers (CC genotype) and carriers (CT genotype) at *APOC3* R19X (x-axis). Although the mean triglyceride level in carriers (33 mg/dL; 2.83 standard deviation) was lower compared with the non-carriers (39.51 mg/dL; 18.52 standard deviation), the difference between the two is not statistically significant (two-sided t-test assuming unequal variances; *p* = 0.11)

As expected when stratified by *APOC3* R19X genotype (Fig. 2), the mean TG level in carriers (33 mg/dL; 2.83 standard deviation) was lower compared with the non-carriers (39.51 mg/dL; 18.52 standard deviation). However, the difference was not statistically significant (*p* = 0.11) in this sample of adults with very low TG levels. Similarly, the mean TG level in IVS2 + 1G > A carriers was 32.5 mg/dL (2.12 standard deviation) for first-mentioned TG level.

Previous observational studies have suggested that TG levels are associated with risk of cardiovascular disease [21]. More recently *APOC3* 19X carrier status among other *APOC3* mutations was associated with lower risk of coronary heart disease [16]. Of the two *APOC3* 19X carriers, one had no evidence in the EHR of myocardial infarction, revascularization, or other heart disease. One *APOC3* 19X carrier had evidence in the EHR of all three. A more detailed review of the de-identified EHRs of the two *APOC3* 19X carriers was performed to identify other possible cardiovascular disease risk factors. The female 19X carrier was born in the 1940s, and her EHR contained a medical history significant for remote breast cancer treated with surgery and radiation, controlled hypertension, and overweight (BMI ~ 28 kg/m^2^). This female 19X carrier had never smoked cigarettes, and there was no evidence of coronary artery disease or myocardial infarction in her EHR. A single assessment of lipids was available for this female 19X carrier: low-density lipoprotein cholesterol (LDL-C) 116 mg/dL, high density lipoprotein cholesterol (HDL-C) 52 mg/dL, and TG 35 mg/dL. The female 19X carrier had unweighted and weighted GRS of 33 and 35.39, respectively. The second *APOC3* 19X carrier was a male born in the 1920s. The male 19X carrier had a past medical history of uncontrolled hypertension, was overweight (BMI 27 kg/m^2^), and was a prior smoker. The male carrier had an extensive history of cardiac disease including atrial fibrillation, coronary artery disease with prior coronary artery bypass grafting, myocardial infarction, and ischemic cardiomyopathy (ejection fraction 30%) with heart failure. The male 19X carrier was not treated with statins due to reported intolerance, and a single measurement of lipids was available: LDL-C 69 mg/dL, HDL-C 41 mg/dL, and TG 31 mg/dL. The male 19X carrier had unweighted and weighted GRS of 32 and 34.21, respectively. Neither *APOC3* 19X carrier has died as of 2015.

The male 19X carrier was also a carrier for IVS2 + 1G > A, the only potentially compound heterozygote described in the literature to date. The other IVS2 + 1G > A carrier was a female born in the 1950s. The female IVS2 + 1G > A carrier was normal weight (BMI 23 kg/m^2^) with two mentions of TG levels (34 and 23 mg/dL) in the EHR. This female IVS2 + 1G > A carrier has no evidence of myocardial infarction, revascularization, or other heart disease in the EHR.

## Discussion

We evaluated 184 adult European American patients with very low TG levels extracted from EHRs for the presence of the loss-of-function allele (19X) for *APOC3* rs76353203. Overall, we identified two carrier patients, and as hypothesized, the resulting allele frequency of *APOC3* 19X was higher in this extreme patient population compared with the general population [10]. Neither carrier patient had the lowest TG levels among this patient population. A review of the EHR revealed only one of the two *APOC3* 19X carriers was free of myocardial infarction, revascularization, and other heart disease. Coincidentally, the *APOC3* 19X carrier with evidence of cardiovascular disease was also a carrier of IVS2 + 1G > A, representing to our knowledge potentially the first compound heterozygote for these mutations in the literature [16].

Based on the current literature [16], we would expect that the addition of these genomic data to the EHR would assist a physician in the assessment of the carriers’ risk of future cardiovascular disease. *APOC3* R19X was originally identified in a genome-wide association study (GWAS) of TG levels in the Pennsylvania Amish where a variant in linkage disequilibrium with R19X was significantly associated with decreased TG levels [8]. Follow-up sequencing revealed the loss of function mutation in *APOC3* likely responsible for the GWAS findings [8], and subsequent studies in both isolated [17, 18] and outbred [10, 16] populations have confirmed the strong association between lower TG levels and 19X carriers. More recently, prospective epidemiologic studies have demonstrated that 19X carriers have lower rates of cardiovascular disease compared with non-carriers [16].

Interestingly, one of the two *APOC3* 19X carriers identified here has evidence in the EHR of a myocardial infarction, revascularization, and other heart disease. Also, apart from sharing low TG levels, the two 19X carriers had different cardiovascular risk profiles. While the statistical evidence for the association between lower TG levels and lower risk of coronary heart disease is strong at the population level, these data highlight the difficulty in translating a genetic association finding in the clinic for risk prediction at the patient level as envisioned for precision medicine. These data also highlight the genetic and environmental heterogeneity that drives cardiovascular disease risk. Thus, the addition of *APOC3* R19X in a clinical setting may contribute to the patient’s risk assessment for cardiovascular disease, but it is not absolute and must be considered with other genetic and environmental risk factors [22].

We specifically targeted adult European Americans with low TG levels for *APOC3* R19X genotyping. The loss-of-function variant is common in isolated populations such as the Pennsylvania Amish [8] and Greek isolates [17, 18], but a study in NHANES confirmed the mutation is rare in the general population [10]. The frequency of 19X also varies by race/ethnicity. In NHANES [10] and in other studies [16], 19X is exceedingly rare in African Americans or African-descent populations compared with European-descent populations. In the present study of European Americans with low TG levels, we observed that the frequency of 19X was higher in this patient population compared with the general population, an observation consistent with the known genetic epidemiology of this loss-of-function variant. Although the carriers identified in this study had lower mean TG levels compared with non-carriers, we did not observe a statistically significant association most likely due to the fact that all patients genotyped already had low TG levels. Also, we only identified two carriers resulting in low statistical power.

The strategy of genotyping or targeting individuals with extreme phenotypes has been a popular and successful strategy in genetic epidemiology for gene discovery for many years [23]. Indeed, in the field of cardiovascular genetics, sequencing individuals with extreme LDL-C, HDL-C, and TG levels in multiple populations has identified several genetic variants and potential drug targets such as *PCSK9* [24]. An analogous strategy could be implemented in a clinical setting to augment the EHR with specific genotypes for an individual patient’s risk assessment. For example, if a patient presents with an extreme lipid level, a panel of known functional variants (missense and loss-of-function) could be ordered for genotyping and added to the EHR to inform risk assessment for future cardiovascular events in that patient. A major advantage of this targeted approach is that the genetic assays would be ordered only on a fraction of the patients (e.g., patients with extreme labs) making it cost-effective compared with offering the panel to all patients regardless of lab results.

There are major disadvantages to a targeted approach to augmenting the EHR with genomic data. For most human traits and diseases, the known functional or strongly associated variants were discovered in European-descent populations [25, 26]. As such, diverse populations such as African Americans and Hispanics may not benefit from a European-centric genotyping panel. And, even among European-descent populations the catalog of genotype-phenotype associations is far from complete or strongly predictive of clinical events [27]. As technology improves to sort functional from neutral variants [28, 29], all patients may benefit from whole genome sequencing.

## Conclusions

Further work is needed in developing appropriate tools for EHR integration and delivery of clinical decision support [30] for this and other clinically relevant genetic variants as envisioned in an era of precision medicine.

## Additional file

Additional file 1: **Table S1.** Genetic variants previously associated with risk of coronary artery disease in European Americans. (DOCX 27 kb)

## Abbreviations

BMI: Body mass index
CAD: Coronary artery disease
CHD: Coronary heart disease
CPT: Current procedural terminology
EHR: Electronic health record
GRS: Genetic risk score
GWAS: Genome-wide association study
HDL-C: High density lipoprotein cholesterol
ICD-9-CM: International Classification of Diseases, Ninth Revision, Clinical Modification
LDL-C: Low-density lipoprotein cholesterol
MAF: Minor allele frequency
NHANES: National Health and Nutrition Examination Survey
TG: Triglycerides
VANTAGE: Vanderbilt Technologies for Advanced Genomics
VUMC: Vanderbilt University Medical Center
