# Supplementary material for: Frequency and phenotype consequence of APOC3 rare variants in patients with very low triglyceride levels
Source: BMC Med Genomics. 2018 Sep 14;11(Suppl 3):66. doi: 10.1186/s12920-018-0387-1 (PMC6156840; doi:10.1186/s12920-018-0387-1)
Supplement: Supplementary file 1 — Table S1. Genetic variants previously associated with risk of coronary artery disease in European Americans. (DOCX 27 kb) [file 12920_2018_387_MOESM1_ESM.docx]

**Table S1. Genetic variants previously associated with risk of coronary artery disease in European Americans. A total of 46 common genetic variants from a genome-wide association study of incident myocardial infarction cases and controls in CHARGE [1] were considered in determining genetic risk scores (GRS) in this patient population. Shown are the SNP ID (rs numbers), coded allele, combined odds ratios, and combined p-values from Tables 1 and 2 of a genome-wide association study of cases and controls of coronary artery disease in CARDIoGRAM [2]. Of the 46 SNPs considered, 37 were directly assayed by the Illumina** HumanExome BeadChip. The nine SNPs not assayed by the HumanExome BeadChip **are denoted by an asterisk. Weighted GRS scores are based on pooled odds ratios from CARDIoGRAM [2].**

| SNP | Coded allele | Odds ratio | p-value |
| --- | --- | --- | --- |
| rs10947789 | T | 1.06 | 1.22x10-5 |
| rs11203042 | T | 1.04 | 6.08x10-6 |
| rs11206510 | T | 1.06 | 1.79x10-5 |
| rs1122608 | G | 1.1 | 6.33x10-14 |
| rs11556924* | C | 1.09 | 6.74x10-17 |
| rs12190287* | C | 1.07 | 4.94x10-13 |
| rs12205331 | C | 1.04 | 4.18x10-5 |
| rs12413409 | G | 1.1 | 6.26x10-8 |
| rs12539895* | A | 1.08 | 5.33x10-4 |
| rs12936587 | G | 1.06 | 4.08x10-10 |
| rs1333049 | C | 1.23 | 1.39x10-52 |
| rs15563 | C | 1.04 | 9.37x10-5 |
| rs1561196* | A | 1.05 | 2.57x10-6 |
| rs17114036 | A | 1.11 | 5.8x10-12 |
| rs17464857 | T | 1.05 | 6.06x10-5 |
| rs17514846 | A | 1.05 | 7.35x10-7 |
| rs2047009* | C | 1.05 | 1.59x10-9 |
| rs2048327 | G | 1.06 | 6.86x10-11 |
| rs2075650 | G | 1.11 | 5.86x10-11 |
| rs2246833* | T | 1.06 | 9.49x10-6 |
| rs2252641 | G | 1.04 | 1.27x10-4 |
| rs2281727 | C | 1.05 | 7.83x10-9 |
| rs2505083 | C | 1.06 | 1.35x10-11 |
| rs264 | G | 1.05 | .70x10-4 |
| rs273909 | C | 1.09 | 2.00x10-7 |
| rs2895811 | C | 1.06 | 4.08x10-10 |
| rs3184504* | T | 1.07 | 5.44x10-11 |
| rs3217992 | A | 1.16 | 7.75x10-57 |
| rs4252120 | T | 1.06 | 1.82x10-5 |
| rs445925 | C | 1.13 | 8.76x10-9 |
| rs4773144 | G | 1.07 | 1.43x10-11 |
| rs4845625 | T | 1.04 | 3.46x10-5 |
| rs501120 | A | 1.07 | 1.79x10-9 |
| rs515135 | G | 1.08 | 2.17x10-8 |
| rs579459 | C | 1.07 | 2.66x10-8 |
| rs602633 | C | 1.12 | 1.47x10-25 |
| rs6725887* | C | 1.12 | 1.16x10-15 |
| rs7173743 | T | 1.07 | 6.74x10-13 |
| rs7692387 | G | 1.06 | 1.89x10-5 |
| rs9319428 | A | 1.05 | 5.70x10-6 |
| rs9326246 | C | 1.09 | 1.51x10-7 |
| rs9369640 | A | 1.09 | 7.53x10-22 |
| rs9515203* | T | 1.08 | 5.85x10-12 |
| rs9818870 | T | 1.07 | 2.62x10-9 |
| rs974819 | A | 1.07 | 3.55x10-11 |
| rs9982601 | T | 1.13 | 7.67x10-17 |

**References**

1. Dehghan A, Bis JC, White CC, Smith AV, Morrison AC, Cupples LA, et al. Genome-Wide Association Study for Incident Myocardial Infarction and Coronary Heart Disease in Prospective Cohort Studies: The CHARGE Consortium. PLOS ONE. 2016;11(3):e0144997. doi: 10.1371/journal.pone.0144997. PMC4780701.

2. Deloukas P, Kanoni S, Willenborg C, Farrall M, Assimes TL, Thompson JR, et al. Large-scale association analysis identifies new risk loci for coronary artery disease. Nat Genet. 2013;45(1):25-33. doi: 10.1038/ng.2480. PMC3679547.
